# SOCS and Herpesviruses, With Emphasis on Cytomegalovirus Retinitis

**DOI:** 10.3389/fimmu.2019.00732

**Published:** 2019-04-11

**Authors:** Christine I. Alston, Richard D. Dix

**Affiliations:** ^1^Department of Biology, Viral Immunology Center, Georgia State University, Atlanta, GA, United States; ^2^Department of Ophthalmology, Emory University School of Medicine, Atlanta, GA, United States

**Keywords:** suppressor of cytokine signaling, SOCS1, SOCS3, herpesvirus, cytomegalovirus, retinitis

## Abstract

Suppressor of cytokine signaling (SOCS) proteins provide selective negative feedback to prevent pathogeneses caused by overstimulation of the immune system. Of the eight known SOCS proteins, SOCS1 and SOCS3 are the best studied, and systemic deletion of either gene causes early lethality in mice. Many viruses, including herpesviruses such as herpes simplex virus and cytomegalovirus, can manipulate expression of these host proteins, with overstimulation of SOCS1 and/or SOCS3 putatively facilitating viral evasion of immune surveillance, and SOCS suppression generally exacerbating immunopathogenesis. This is particularly poignant within the eye, which contains a diverse assortment of specialized cell types working together in a tightly controlled microenvironment of immune privilege. When the immune privilege of the ocular compartment fails, inflammation causing severe immunopathogenesis and permanent, sight-threatening damage may occur, as in the case of AIDS-related human cytomegalovirus (HCMV) retinitis. Herein we review how SOCS1 and SOCS3 impact the virologic, immunologic, and/or pathologic outcomes of herpesvirus infection with particular emphasis on retinitis caused by HCMV or its mouse model experimental counterpart, murine cytomegalovirus (MCMV). The accumulated data suggests that SOCS1 and/or SOCS3 can differentially affect the severity of viral diseases in a highly cell-type-specific manner, reflecting the diversity and complexity of herpesvirus infection and the ocular compartment.

## Introduction

Herpesviruses skillfully manipulate their hosts by various mechanisms while viral lytic and latent cycles maintain a lifelong, Sisyphean struggle with host innate, and adaptive immune systems. Cells of innate and adaptive immunity are efficient producers of pro-inflammatory cytokines, chemokines, and cell surface receptors, and they rely heavily on cell-type-specific intracellular signaling pathways to differentiate and function properly. Upon infection, herpesviruses are recognized by circulating innate cells such as monocytes, macrophages, dendritic cells (DC), neutrophils, or natural killer (NK) cells (1), and by local resident innate cell types specialized in certain tissues, such as Müller cells and microglia (2) of the retina. Interactions between receptors and pathogens begin signaling cascades that result in progressively amplified, harmonious transcriptional stimulation of hundreds of downstream gene products, many of them cytokines released extracellularly to function in autocrine or paracrine positive feedback capacities. of homeostasis being paramount for biological systems, this signaling also induces negative feedback agents such as suppressor of cytokine signaling (SOCS) proteins to aid in the prevention of damaging immunopathologies. The eight known SOCS members comprise a family of host proteins which, among their other functions, negatively regulate signaling pathways induced by antiviral and inflammatory cytokines, effectively increasing tolerance for specific cytokines signaling within specific cells [for reviews, see (3–6)]. Once activated, innate immune cells such as DCs or microglia can become professional antigen presenting cells, which instruct and activate adaptive immune cells such as B cells and CD4^+^ and CD8^+^ T lymphocytes to produce their effector functions against pathogens and pathogen-infected cells. During primary and lytic infection, herpesviruses nimbly evade sufficient aspects of innate and adaptive immunity to avoid complete clearance. Eventually they enter or are forced by the immune system into a state of latency during which the virus continues to modulate host immunity despite only a small subset of viral genes being detectable. Reactivation from latency to lytic infection then back to latency may then occur periodically throughout the life of the host [for reviews, see (1, 7–10)].

Despite the relatively large number of virus-encoded gene products contained within herpesviruses compared with other viruses, they remain obligate intracellular pathogens and therefore still rely on host-encoded gene products for survival and propagation. SOCS proteins are one such example of host-encoded proteins that are manipulated by many different types of viruses and other pathogens, as reviewed by others (5, 6). In addition to the viruses featured in these reviews, more herpesviruses also are now known to stimulate SOCS1 and/or SOCS3 during *in vitro* or *in vivo* infection. These include the human herpesviruses herpes simplex type 1 (HSV-1), varicella zoster virus (VZV), human cytomegalovirus (HCMV), Epstein-Barr virus (EBV), and Kaposi's sarcoma-associated herpesvirus (KSHV), as well as the animal herpesviruses *gallid alphaherpesvirus 2* (GaHV-2, or Marek's disease virus, MDV), *suid alphaherpesvirus 1* (SuHV-1, or pseudorabies virus, PRV), murine cytomegalovirus (MCMV), and murine gammaherpesvirus-68 (MHV-68) (11–23).

Herein we discuss these human and animal herpesviruses currently known to affect SOCS proteins in various *in vitro* and *in vivo* model systems, with particular emphasis on SOCS1 and SOCS3 expression during experimental MCMV retinitis, a mouse model used to study AIDS-related HCMV retinitis (24). AIDS-related HCMV retinitis is a blinding, degenerative disease of the retina that once threatened the bilateral vision of ~30% of AIDS patients (25). Despite the advent of antiretroviral therapies (ART) in the developed world, HCMV remains a significant opportunistic pathogen of AIDS patients worldwide. As with humans and AIDS, mice with murine AIDS (MAIDS) experience retrovirus-induced immune suppression and become susceptible to diseases of opportunistic pathogens (26). For many years our laboratory has used MAIDS-related MCMV retinitis as a clinically relevant mouse model with high face validity and predictive validity [per (27, 28)] to AIDS-related HCMV retinitis to elucidate the role of potential candidates contributing to this disease (29), including host SOCS proteins (21, 23). Thus, the purposes of this review are to explore briefly the model systems under which herpesviruses manipulate SOCS proteins and to review the effects of SOCS manipulation on virologic, immunologic, or pathologic outcomes, with a focus on experimental cytomegalovirus retinitis. Specialized therapeutic inhibition or mimicry of SOCS proteins, perhaps combined with immunotherapies or antiviral drugs, may become a viable tactic for more effectively combating herpesvirus pathologies.

## Suppressor of Cytokine Signaling (SOCS) Family

Innate and adaptive immune cells secrete cytokines and chemokines to orchestrate a coherent, integrated immune response to protect the host against pathogens. During infection, cytokines initiate, execute, and resolve inflammatory responses, such that cytokine signaling is the crucial control switch between the initiation of the immune response and the maintenance of homeostasis in the periphery. Therefore, cellular negative feedback loops play an important role in maintaining the tight balance of cytokine secretion and cytokine inhibition, and SOCS proteins function in such a capacity.

### SOCS Structure, Function, and Expression

**Figure 1:**
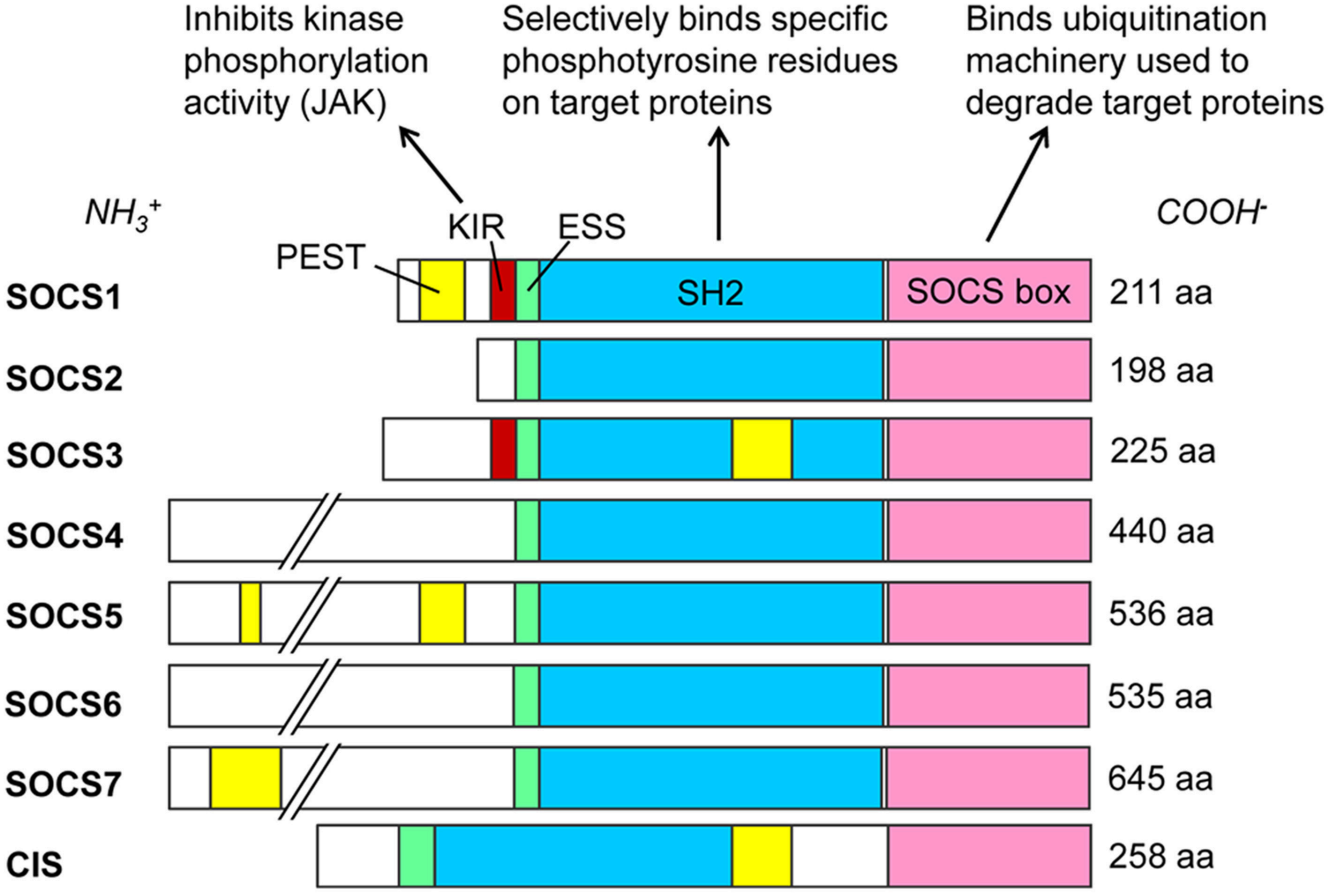
SOCS family proteins and their domains. Src homology 2 (SH2) domains (blue) govern target protein specificity by recognizing phosphorylated tyrosine residues flanked by specific sequences such as those on cytoplasmic residues of cytokine receptors. SOCS1 and SOCS3 exclusively contain kinase inhibitory regions (KIR, red), which bind and inhibit JAK proteins. Extended SH2 sequences (ESS, green) enhance binding specificity and affinities to phosphotyrosine residues. SOCS box domains (pink) recruit cellular Elongin BC, Cullin5, and RING-box-2 to form an E3 ubiquitin ligase complex, ubiquitinating target proteins for proteasomal degradation. PEST motifs (yellow) greatly decrease the half-lives of the proteins; see (35, 36) for predicted PEST domain locations. Amino acid (aa) lengths for *Homo sapiens* SOCS proteins are from the National Center for Biotechnology Information (NCBI) database (February 2019).

SOCS proteins were first discovered in the mid-1990s as cytokine-induced inhibitors of signal transducers and activators of transcription (STAT) cell signaling pathways (30–33). The SOCS protein family currently contains eight known members: SOCS1 through SOCS7 and the cytokine-inducible Src homology 2 (SH2)-containing domain protein (CIS). These proteins are selectively upregulated in response to various cell signaling pathways (34) and subsequently act intracellularly as negative regulators of cell signaling (4). All SOCS proteins characteristically contain a C-terminal SOCS box, an internal SH2 domain, and a variable-length N-terminal region (4) (Figure 1). SH2 domains are conserved throughout most eukarya, excluding single-celled fungi, and they recognize and bind to specific phosphorylated tyrosine motifs on their target proteins (37). At least 110 unique human proteins contain SH2 domains (38), and specificity to their targets is achieved by primary and secondary binding sites within these SH2 domains (39). Immediately upstream of the SH2 domain is the extended SH2 sequence (ESS) which increases binding affinity to phosphotyrosine residues (40–42). The SOCS box is also a conserved sequence found within more than 70 different human proteins (43). This motif primarily functions to recruit cellular ubiquitination machinery, thus allowing such proteins to flag their specific substrates for proteasomal degradation (43). It achieves this by binding cellular Elongin B, Elongin C, Cullin5, and RING-box-2, thus forming an E3 ubiquitin ligase complex (4–6, 43). SOCS1 and SOCS3 additionally possess an N-terminal kinase inhibitory region (KIR) which can act as a pseudosubstrate to block the kinase activity of such proteins as Janus kinases (JAKs) (32, 44, 45). These SOCS proteins negatively regulate intracellular signaling pathways by several mechanisms, including competitive binding of phosphotyrosine residues with various recruited STAT proteins, inhibition of JAK activity via KIR domains, or ubiquitination of SOCS-bound elements by the SOCS box, marking them for degradation (4, 5). In addition to these domains, SOCS1, SOCS3, SOCS5, SOCS7, and CIS each contain a sequence rich in proline (P), glutamic acid (E), serine (S), and threonine (T) known as a PEST motif (46), which decreases the half-life of the entire protein to about 2 h (42). The predicted locations for these PEST motifs vary, and to our knowledge no such predicted sequence has yet been found for SOCS2, SOCS4, or SOCS6 (35, 36).

**Figure 2:**
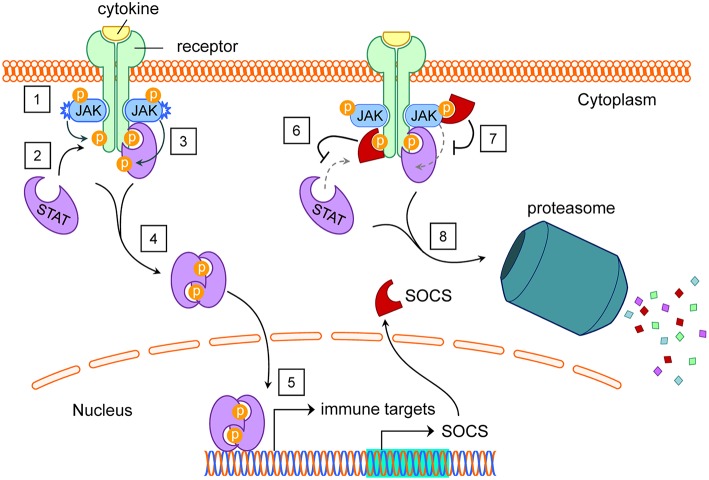
SOCS induction by and inhibition of the JAK/STAT pathway. **(1)** Extracellular cytokines cause dimerization of their cognate transmembrane receptors. This brings intracellular receptor-associated JAK proteins into proximity to cross-phosphorylate each other and tyrosine residues on the receptors. **(2)** STAT proteins dock at phosphotyrosines on intracellular receptor subunits. **(3)** JAK proteins phosphorylate STAT proteins, activating them. **(4)** Activated STAT proteins undock from their receptors, dimerize, and translocate to the nucleus. **(5)** STAT proteins act as transcription factors for dozens of immune targets, including SOCS. **(6)** Functioning in the cytoplasm, SOCS proteins can bind various phosphotyrosines on intracellular receptors, blocking STATs from their native docking sites. **(7)** With their KIR domains, SOCS1 and SOCS3 can inhibit the kinase activity of JAK proteins, preventing tyrosine phosphorylation of STAT proteins. **(8)** SOCS boxes facilitate ubiquitination of SOCS-bound protein targets for proteasomal degradation. Abbreviations: suppressor of cytokine signaling (SOCS), Janus kinase (JAK), signal transducers and activators of transcription (STAT), kinase inhibitory region (KIR). See Akhtar and Benveniste (5).

Several different types of cell signaling pathways are capable of inducing SOCS (47–50), with JAK/STAT signaling driven by cytokines such as interferons (IFN) and interleukins (IL) being one of the best studied SOCS-inducing pathways (4). When transmembrane cytokine receptors on a cell surface recognize their cognate extracellular cytokines, they initiate intracellular phosphorylation cascades via specific combinations of JAK and STAT proteins, transcriptionally stimulating scores of gene products (51–53), including negative-feedback SOCS family proteins. Well-described cytokine receptor-JAK/STAT-gene target combinations are reviewed and summarized elsewhere (54, 55). Intracellular SOCS proteins then selectively inhibit components of JAK/STAT and other cell signaling pathways, within the specific cells expressing them (4, 33, 56–58) (Figure 2). Although some crosstalk occurs between individual SOCS members and their targets, the variations between SOCS protein SH2 domains equip them with preferential affinity to their respective substrates, as listed elsewhere (50). Receptor expression, cytokine milieus, and signaling pathways tend to differ greatly between cell types, even within the contexts of different tissues or microenvironments.

Many different cell types in various organs are capable of producing SOCS family proteins (33), and they are most amply produced by hematopoietic cells (59) of the innate and adaptive immune systems (4, 58). Some of these SOCS-expressing cell types include monocytes (60), macrophages (32, 61), DCs (62, 63), microglia (64), neutrophils (65), NK cells (66), CD4^+^, and CD8^+^ T cells (67, 68), and ocular Müller cells (69). SOCS proteins primarily function within the very cells which transcriptionally produce them, although cell-to-cell vesicular transport of SOCS proteins has been demonstrated from alveolar macrophages to adjacent epithelial cells (70).

### SOCS1 and SOCS3

The importance of SOCS1 and SOCS3 in modulating immune responses is emphasized in knockout mice, as SOCS1-deficient mice die within 3–4 weeks of birth from massive IFN-related inflammation (71–73), and deletion of the SOCS3 gene is embryonically lethal (74). SOCS1 proteins are able to limit the surface expression of molecules that mediate the immune response, suppress inflammation by dampening expression of cytokines and chemokines, inhibit pathogen infiltration and replication, and prevent central nervous system demyelination. SOCS1 is quickly induced by IFN signaling and inhibits the specific JAK and STAT proteins involved during IFN signaling (75, 76). In addition to its primary role in the regulation of components of the JAK/STAT pathway, SOCS1 is capable of regulating other cellular signaling pathways such as toll-like receptor (TLR) signaling and macrophage activation (47). Whereas inactivated macrophages produce low baseline levels of SOCS1 and SOCS3, induction of SOCS1 generally drives macrophages toward an M2 phenotype, and SOCS3 toward M1 (77, 78). SOCS1 also plays a dual role in CD4^+^ T-helper (T_H_) cell differentiation (67, 79–81). As a key attenuator of type II IFN (IFN-γ) signaling, SOCS1 can inhibit IFN-γ-mediated STAT1 activation by targeting JAK2, thus suppressing the differentiation of the T_H_1 lineage in CD4^+^ T cells (75, 82). SOCS1 is alternatively able to inhibit IL-4 signaling, thereby driving differentiation toward a T_H_1 phenotype (67, 83). By comparison, SOCS3 is classically upregulated as a consequence of signaling by the IL-6 family of cytokines (33). Once induced, a major function of SOCS3 is then to inhibit the signaling of IL-6 family cytokines by targeting their common gp130 receptor (58, 84, 85). Furthermore, SOCS3 is a key regulator of IL-23-mediated STAT3 (79, 86) and of IL-12-mediated STAT4 activation (85), such that SOCS3 is also able to inhibit the development of CD4^+^ T_H_1 and T_H_17 cells (87), thereby promoting differentiation to the T_H_2 lineage.

Both SOCS1 and SOCS3 have demonstrated transcriptional induction by type I IFNs, key immune regulators in mounting an antiviral response (88, 89). These cytokines play a role in the activation of NK and T cells, and they induce cell death in virus-infected cells (90, 91). The type I IFN family consists of the many subtypes of IFN-α, as well as IFN-β, IFN-ε, IFN-κ, and IFN-ω (92). Almost all cell types are capable of producing type I IFNs in response to various stimuli (89, 90, 93). Plasmacytoid DCs (pDC) in particular are one of the highest contributors to the secretion of type I IFNs (90). Type I IFNs signal through the heterodimerization of the type I IFN receptor (IFNAR)-1 and IFNAR-2, which signal through the JAK/STAT pathway, mediated specifically by the JAKs Tyk2 and JAK1, and by STAT1, and STAT2 (90, 94). Unlike most dimerized STATs, the STAT1/STAT2 heterodimer must bind to an additional protein, interferon regulatory factor 9 (IRF9), and form the interferon-stimulated gene factor 3 (ISGF3), before they are able to recognize the interferon-stimulated response element (ISRE) and begin transcription of ISGs (90). The more than 300 ISGs that have been identified to date (95) include SOCS proteins, particularly SOCS1, and, to a lesser extent, SOCS3.

In addition to this classical induction by cytokine signaling via the JAK/STAT pathway, SOCS proteins have also shown to be stimulated by alternative cell signaling pathways. Among these pathways are nuclear factor κB (NF-κB) and mitogen activated protein kinase (MAPK) signaling pathways through phosphorylation of c-Jun N-terminal kinases (JNKs) (96, 97). SOCS proteins can also be induced by stimulation of TLRs (48, 98, 99), which are expressed by many cell types, including the retinal pigment epithelium (RPE) (100, 101) and Müller cells (102) of the eye. In macrophages and DCs, non-TLR sensor dectin-1 induces SOCS1 by MAPK/ERK, and SOCS1 modulates TLR9 signaling by inhibiting NF-κB (103). Stimulation of these pathways therefore may trigger the production of SOCS proteins directly or indirectly by the production of SOCS-inducing cytokines such as type I IFN.

### SOCS2

Although the rest of the SOCS family (CIS, SOCS2, and SOCS4—SOCS7) remains less studied than SOCS1 and SOCS3, ever more research on these accumulates over time. SOCS2, briefly discussed below in the context of alphaherpesviruses, is stimulated within different cell types in response to signals from various hormones or cytokines, including growth hormone, insulin, IFN-α, and IL-6, possibly through STAT5 [reviewed in (6, 104)]. It is believed that SOCS2 and CIS primarily bind to phosphotyrosines on intracellular receptor residues to block STAT binding in a competitive manner (5). Among its other functions, SOCS2 negatively regulates the growth hormone receptor, and SOCS2-knockout mice are significantly (~40%) larger than wild type mice (105). Like most other SOCS members, SOCS2 is also implicated in some types of cancer, albeit less abundantly so than other SOCS members.

## Herpesviruses

### *Herpesviridae* Classification and Characteristics

Admittance into the *Herpesviridae* family of the taxonomic order *Herpesvirales* traditionally is based upon the virus structure: dsDNA within an icosahedral capsid surrounded by an amorphous tegument between the host cell-derived envelope encrusted with viral glycoproteins. Members of this family share the biological characteristics of replication within host cell nuclei, the establishment of latency, and ultimate destruction of lytically infected host cells. With notable exceptions, it is generally rare that herpesviruses cause severe disease in immunocompetent, endogenous hosts, with the majority of morbidities or mortalities occurring in the very young, very old, immune compromised, or non-native host. To date, there are nine known herpesviruses that infect humans; these are designated human herpesvirus (HHV)-1 through HHV-8, with a ninth member in the division of HHV-6 into HHV-6A and HHV-6B (106) as distinct herpesvirus species. The *Herpesviridae* family contains three subfamilies: *Alphaherpesvirinae, Betaherpesvirinae*, and *Gammaherpesvirinae*. Members of these subfamilies are phylogenetically classified based on genetic sequence homology but can also be generally distinguished by their respective cell or tissue preference for establishing latency, relative rate of replication cycle, and/or natural or experimental host restriction [reviewed in (1, 107–110)]. Classifications of select herpesviruses pertinent to this review are organized in Table 1.

**Table 1:** Taxonomic classifications of select members of the *Herpesviridae* family.

	**Genus**	**Species name**	**Common name**
α	*Mardivirus*	*Gallid alphaherpesvirus 2* (GaHV-2)	Marek's disease virus (MDV)
	*Simplexvirus*	*Human herpesvirus 1* (HHV-1)	Herpes simplex virus type 1 (HSV-1)
		*Human herpesvirus 2* (HHV-2)	Herpes simplex virus type 2 (HSV-2)
	*Varicellovirus*	*Bovine herpesvirus 1* (BoHV-1)	
		*Bovine herpesvirus 5* (BoHV-5)	
		*Human herpesvirus 3* (HHV-3)	Varicella zoster virus (VZV)
		*Suid herpesvirus 1* (SuHV-1)	Pseudorabies virus (PRV)
β	*Cytomegalovirus*	*Human herpesvirus 5* (HHV-5)	Human cytomegalovirus (HCMV)
	*Muromegalovirus*	*Murid herpesvirus 1* (MuHV-1)	Murine cytomegalovirus (MCMV)
	*Roseolavirus*	*Human herpesvirus 6A* (HHV-6A) *Human herpesvirus 6B* (HHV-6B)*Human herpesvirus 7* (HHV-7)	
γ	*Lymphocryptovirus*	*Human herpesvirus 4* (HHV-4)	Epstein-Barr virus (EBV)
	*Rhadinovirus*	*Human herpesvirus 8* (HHV-8)	Kaposi's sarcoma-associated herpesvirus (KSHV)
		*Murid herpesvirus 4* (MuHV-4), isolate MHV-68	Murine gammaherpesvirus 68 (MHV-68)

*Herpesviridae subfamilies: Alphaherpesvirinae (α), Betaherpesvirinae (β), Gammaherpesvirinae (γ). Classifications from the July 2017 International Committee on Taxonomy of Viruses (ICTV) and Pellett and Roizman (1) and Davison et al. (109)*.

#### Alphaherpesvirinae

The α-herpesviruses are characterized by their ability to establish latency in neurons, to infect a variety of host species, to replicate and spread relatively quickly, and to destroy infected host cells. This subfamily currently consists of five genera, two of which infect mammals: *Simplexvirus* and *Varicellovirus*. Pathologies of *Simplexvirus* HSV-1 include oropharyngeal lesions (cold sores), herpetic epithelial or stromal keratitis, herpes simplex encephalitis, and genital herpes (111), with the latter more frequently caused by HSV-2, another *Simplexvirus*. VZV of the *Varicellovirus* genus is the etiological agent of varicella (chickenpox) and herpes zoster (shingles). Also in this genus is *suid alphaherpesvirus 1* (SuHV-1), or PRV, which causes fatal disease following natural infection of swine as well as a wide range of mammalian host species. In addition, *bovine herpesvirus 1* (BoHV-1) and BoHV-5 are highly similar varicelloviruses (112) which cause significant infections of cattle (113, 114). The genus *Mardivirus* of the *Alphaherpesvirinae* subfamily contains *gallid alphaherpesvirus 2* (GaHV-2), or MDV, which infects chickens and is responsible for significant losses in the poultry industry (115, 116).

#### Betaherpesvirinae

The β-herpesviruses generally replicate more slowly than other herpesviruses and display host species specificity, with a propensity to establish latency in lymphoid cells of hematopoietic origin. The genus *Roseolavirus* comprises HHV-6 and HHV-7, of which HHV-6B and HHV-7 have been shown to cause exanthem *subitum* (roseola, sixth disease) (106, 117). Of particular importance to this review are the genera *Cytomegalovirus*, which contains HCMV, and *Muromegalovirus*, which includes *murid herpesvirus 1* (MuHV-1), or MCMV. HCMV and MCMV represent a central focus of this report and are discussed more thoroughly in following sections.

#### Gammaherpesvirinae

The γ-herpesvirus subfamily contains viruses that are species-specific, generally prefer B or T lymphocytes for replication, and establish latency within lymphoid tissue. This subfamily contains four genera, of which *Lymphocryptovirus* contains EBV, and *Rhadinovirus* includes KSHV (HHV-8) and MHV-68, an isolate of *murid herpesvirus 4* that is widely used in experimental model systems (1).

### Herpesvirus Immune Evasion: HCMV and MCMV

The balance between virulence and the host immune response sways the outcome of any viral infection. Just as the host has an arsenal of mechanisms for sensing, stopping, and clearing viral infection, viruses have as many mechanisms for evading, escaping, and producing productive infections in the host. Herpesviruses undergo lytic and latent life cycles for the lifetime of their hosts, and they are particularly adept at manipulating the innate and adaptive immune responses by a multitude of mechanisms. As HSV-1 is a quintessential example of the α-herpesviruses, HCMV and its mouse counterpart MCMV are well-studied examples of the β-herpesviruses. HCMV and MCMV, like many herpesviruses, modulate their host cells by interfering with signaling pathways important to the innate or adaptive immune response (110). As HCMV and MCMV represent a major focus of this review, they are depicted in this section as examples of herpesvirus immune evasion.

Integral to the first-responding cells of innate immunity is the vast family of pattern recognition receptors (PRR) which are capable of detecting common non-self, pathogen-associated molecular patterns (PAMPs) (118). PAMPs are highly-conserved molecules which are usually indispensable to the pathogens with which they are associated (91, 118, 119). Many types of PRRs have been identified so far, including TLRs, retinoic acid-inducible gene I (RIG-I)-like receptors (RLRs), nucleotide oligomerization domain (NOD)-like receptors (NLRs), C-type lectin receptors (CLRs), and absent in melanoma 2 (AIM2)-like receptors (120, 121). In general, activation of any of these PRRs leads to one or more well-characterized cell signaling pathways responsible for the upregulation of pro-inflammatory cytokines, including type I IFNs (120). Among these pathways are NF-κB and MAPK signaling pathways through phosphorylation of JNKs (96, 97), as well as inflammasome/caspase-1-dependent IL-1β maturation (122). Infection with herpesviruses such as HCMV or MCMV has the capacity to stimulate and/or to modulate several of these PRRs (110). For instance, MCMV infection of monocytes and other cell types stimulates TLR2/myeloid differentiation primary response 88 (MyD88) (123), TLR3/TIR-domain-containing adapter-inducing interferon-β (TRIF), and TLR9/MyD88 (124) signaling. Macrophages and their progenitor cells (monocytes, bone marrow cells) highly express PRRs and are major players during systemic HCMV or MCMV dissemination and latency (125–133).

As major players in the innate immune response, macrophages exhibit divergent activation phenotypes in response to various stimuli. These have very generally been categorized into M1 classically-activated macrophages and M2 alternatively-activated macrophages (134), so called for their association with CD4^+^ T_H_1 or T_H_2 polarization, respectively. In general, M1 macrophages are activated via exposure to IFN-γ alone or together with tumor necrosis factor (TNF)-α, PAMPs such as TLR4-recognized lipopolysaccharide (LPS). They express TNF-α, IL-6, IL-1, and IL-12 upon activation, and through production of these pro-inflammatory cytokines and nitric oxide, they exhibit a pro-inflammatory phenotype (135). Alternatively-activated M2 macrophages have grown to include all non-classically-activated macrophages and therefore display a diverse range of activation phenotypes. An M2 phenotype is generally induced by exposure to IL-4 or corticosteroids, results in the production of anti-inflammatory IL-10 and IL-1 receptor antagonist, and participates in anti-inflammatory or pro-angiogenic activities (135). These macrophage polarizations exhibit extreme plasticity, however, and are not as clearly defined as originally thought. Monocytes infected with HCMV, for instance, display a hybrid M1/M2 activation phenotype, simultaneously showing pro-inflammatory and pro-angiogenic properties, but with a propensity mostly toward the M1 phenotype (136–139).

Also integral in early control of herpesvirus infection are NK cells. These granulocytic cells are highly effective at destroying cells that fail to display sufficient amounts of major histocompatibility complex (MHC) class I (MHC-I), which presents intracellularly-derived antigens to MHC-I-restricted immune cells such as CD8^+^ T cells (140). The cytotoxic effector function of NK cells also requires signaling by activating receptors and/or signaling by cytokines such as type I IFN or IL-12 (141). Activated NK cells produce high amounts of IFN-γ and use an arsenal of cytotoxic molecules like perforin or granzyme B to fulfill their cytotoxic functions (142). NK cells play a protective role in response to systemic HCMV and MCMV infections (110, 143) and are primarily responsible for immediate control of infection.

In addition to the immediate response of NK cells of the innate immune system, large numbers of MHC-II-restricted CD4^+^ T cells as well as MHC-I-restricted CD8^+^ T cells of the adaptive immune system specifically target HCMV or MCMV antigens during viral infection (110, 142, 144). More so than the HCMV- or MCMV-specific antibody response of B cells, T cells keep the virus in check throughout the life of the host and play a role in the balance between persistent infection and latency (141, 142). The importance of CD4^+^ and CD8^+^ T cells in controlling lifelong HCMV or MCMV infection is underscored by the profound susceptibility to cytomegalovirus-derived pathologies that occur during depletion or dysfunction of these cells (24, 25, 107, 108, 110, 145–152).

## Herpesviruses Affecting Host SOCS Proteins

Because of the immunomodulatory effects of SOCS proteins, it is not surprising that infectious microbes may take advantage of host SOCS expression. Indeed, SOCS1 and/or SOCS3 exploitation by such viruses as human immunodeficiency virus (HIV) (153–156), hepatitis B virus (157), hepatitis C virus (158, 159), Semliki forest virus (56), respiratory syncytial virus (160), coxsackievirus (161), Ebola virus (162), influenza A virus (163), HSV-1 (164–166), and EBV (12) has been beautifully reviewed elsewhere (5, 6). As Akhtar and Benveniste foresaw, more viruses affecting SOCS proteins have been discovered, many of them herpesviruses. In addition to HSV-1, these include the human herpesviruses VZV (17), HCMV (14), and KSHV (13), as well as the animal herpesviruses MDV (15, 19), PRV (20), MCMV (11, 16, 18, 21, 23), and MHV-68 (22) (Table 2). In addition to these, recent reports discuss the effects of SOCS2 gene knockout during infection with HSV-1 (171), HSV-2 (172), or BHV-5 (173). It is likely that still more viruses affecting SOCS proteins will be discovered in the future.

**Table 2:** Herpesviruses that manipulate host SOCS expression.

**Virus**	**SOCS**	**Cell/tissue type**	**Effect**	**References**
HSV-1	↑SOCS1	HEL-30 (*not L929*), J774A.1 at M0, FL	↓IFN-γ signaling, ↑viral replication	(166–168)
	↑SOCS3	FL, TALL-1, CCRF-CEM (*not U937, THP-1, AKATA*)	↓IFN-α/β signaling, ↑viral replication	(164, 165)
VZV	↑SOCS1	MRC-5, HaCaT		(17)
	↑SOCS3	MRC-5, HaCaT, THP-1	↓IL-6 production, ↑viral gene expression	
HCMV	↑SOCS1 ↑SOCS3	Human MoDC		(14)
EBV	↑SOCS1 ↑SOCS3	HK-1, NP69 PBMC	↓JAK/STAT ↓IFN-α/β positive feedback signaling	(12, 169)
KSHV	↑SOCS3	Primary human endothelial cells	↓neutrophil recruitment ↓IFN-γ/STAT1 signaling, ↓MHC II, CIITA	(13, 170)
MDV	↑SOCS1 ↑SOCS3	Thymus, spleen, and skin of chickens	Unknown	(15, 19)
PRV	↑SOCS3	RAW264.7		(20)
MCMV	↑SOCS1 ↑SOCS3	BMM, IC-21, MEF, mouse eyes during experimental MCMV retinitis	↑Severity retinitis correlation	(11, 18, 21, 23)
MHV-68	↑SOCS1	BMM, RAW264.7 (*but not MLE-12, NIH3T3*)	↓IFN-γ signaling ↑viral replication	(22)

*↑ increases; ↓ decreases. Cells: HEL-30 mouse keratinocytes, L929 mouse fibroblasts, J774A.1 mouse macrophages, FL human amnion cell line, TALL-1 T-cell leukemia cell line CCRF-CEM T-lymphoblastoid cell line, U937 and THP-1 human monocytes, AKATA EBV-negative clone of the Burkitt's lymphoma B-cell line, MRC-5 human lung fibroblasts, HaCaT human keratinocytes, monocyte-derived dendritic cell (MoDC), HK-1 and NP69 human nasopharyngeal epithelial cell lines, primary human peripheral blood mononuclear cells (PBMC), RAW264.7 mouse (BALB/c strain) macrophages, primary mouse bone marrow macrophages (BMM), IC-21 mouse (C57BL/6 strain) macrophages, MLE-12 mouse lung epithelial cells, NIH3T3 mouse fibroblasts*.

### Human Herpesviruses and SOCS1 or SOCS3

The consequences of virally manipulated SOCS1 and SOCS3 expression during HSV-1 infection are probably thus far the best studied among herpesviruses. After hepatitis C virus, HSV-1 is the second virus reported to stimulate host SOCS3 (164). In the human amnion cell line FL (174), this SOCS3 induction occurs very early, within 1 h post-infection (hpi) and coincides with reduction in type I IFN signaling downstream of JAK phosphorylation (164). The same group soon after reported that this is cell-type-specific, as SOCS3 is upregulated within 1 hpi (HSV-1 strain VR3) in the human T-cell leukemia cell line TALL-1 and the T-lymphoblastoid cell line CCRF-CEM, but not in human U937 or THP-1 monocytic cell lines, nor in an EBV-negative clone of the Burkitt's lymphoma B-cell line AKATA (165). This SOCS3 stimulation in FL cells is partly dependent on activation of JAK3 (165). Furthermore, siRNA-targeted suppression of SOCS3 results in lower HSV-1 virus titers in FL cells. Taken together, these studies provide strong evidence that during HSV-1 infection of FL cells, JAK3 signaling stimulates SOCS3, which then modulates the antiviral effects of IFN-α/β signaling, thus facilitating greater viral replication (165). Although this group found no stimulation of SOCS1 within 1 hpi in these cell types with HSV-1 strain VR3, they later detected both SOCS1 and SOCS3 transcriptional stimulation by RT-qPCR in FL cells at 4 hpi that is dependent on the HSV-1 UL13 protein kinase (167). Still others (166) later reported that HSV-1 strain syn17^+^ stimulates SOCS1 expression between 1 and 6 hpi in HEL-30 keratinocytes but not L929 fibroblasts, cell lines derived from mouse strain C3H. Importantly, this correlates with the ability of IFN-γ to protect L929 cells but not HEL-30 cells from HSV-1-induced cell death, with inhibition of STAT1α activation downstream of IFN-γ signaling, and with transcriptional activation of the SOCS1 promoter (166). In primary human astrocytes and neurons, SOCS1 expression during HSV-1 infection is significantly reduced by exposure to type III IFN (IFN-λ) in primary human astrocytes and neurons (175). This cell type specificity for virologic and/or immunologic outcomes is a common theme with herpesviruses, with some outcomes even limited to specific cell activation phenotypes. For instance, HSV-1 infection stimulates SOCS1 in unactivated (M0) J774A.1 mouse macrophages (BALB/cN strain), but not in M1 nor M2 activated macrophages (168).

The α-herpesvirus VZV of the *Varicellovirus* genus initially infects the lungs then disseminates through the blood to cause skin lesions characteristic of varicella (chicken pox). The virus establishes lifelong latency in dorsal root ganglia, where it may reactivate to cause herpes zoster (shingles) (176). Primary infection elicits an innate immune response characterized by stimulation of IFN-α and IFN-γ (17, 176) that is kept in check by multiple viral mechanisms (177). In immunocompetent individuals, adaptive immunity follows, and although anti-VZV antibodies are abundantly produced by B cells, an effective T-cell response is more important for control of severe disease (178), as with many herpesviruses. During experimental *in vitro* infection of permissive cell lines, VZV stimulates SOCS1 and, to a greater extent, SOCS3 in HaCaT human keratinocytes and MRC-5 human lung fibroblasts, and it also stimulates SOCS3 but not SOCS1 in THP-1 human monocytes (17). Suppression of SOCS3 by siRNA significantly reduces viral gene expression and greatly increases IL-6 production during VZV infection of MRC-5 cells (17).

The β-herpesvirus HCMV persistently infects about 80% of the worldwide population without usually causing disease in immunocompetent individuals (110, 179). As with most herpesviruses, most severe HCMV pathologies present only during immune suppression, as in HIV/AIDS patients or solid organ recipients, or underdevelopment of immunity (congenital cytomegalovirus) rather than in immunocompetent hosts. AIDS-related HCMV retinitis, for instance, causes vision loss and blindness in ~30% of untreated AIDS patients (110, 152, 180–183). Upon primary infection, HCMV disseminates via the blood to various organs and establishes latency in circulating monocytes and bone marrow cells (129). Monocyte-derived DCs infected with HCMV (TB40/E or VHLE strains with endothelial cell tropism) stimulate SOCS1 and SOCS3 compared with uninfected cells (14). SOCS3 upregulation in these cells occurs via HCMV stimulation of IL-6/STAT3 signaling, and once stimulated, SOCS3 but not SOCS1 inhibits STAT5 activation downstream of the granulocyte-macrophage colony-stimulating factor (GM-CSF) receptor (14). GM-CSF/STAT5 signaling in monocytic cells drives differentiation toward DCs, and inhibition of this pathway in already-differentiated DCs by HCMV-driven SOCS3 changes their phenotype from CD1a^+^ to CD1a^−^, rendering them inefficient at presenting lipid antigens to T cells (14). Genome sequence analysis of human epithelial HEK293 cells stably expressing the HCMV viral protein US27 showed stimulation of SOCS2 and SOCS5, but not SOCS3, compared with nontransfected HEK293 cells (184), suggesting that the HCMV-encoded G-protein coupled receptor protein US27 may not contribute to SOCS3 stimulation. Like many other herpesviruses, the HCMV genome contains homologs presumably purloined from their hosts (185), such as HCMV-encoded vIL10 (186). HCMV vIL10 stimulates SOCS3 in HeLa cells (187) and monocytes (188). These studies demonstrate pathways whereby HCMV indirectly stimulates SOCS1 and/or SOCS3 in various cell types, which then functionally change host and/or bystander cells to contribute to viral immune evasion.

EBV is a γ-herpesvirus in the genus *Lymphocryptovirus* that ubiquitously infects most of the world's population, frequently without symptoms, and establishes latency in B cells (189). Along with causing most cases of infectious mononucleosis, EBV also is associated with many types of cancer such as nasopharyngeal carcinoma and Burkitt's lymphoma (189, 190). Although the virus efficiently infects B-cell lines *in vitro*, experimental infection of epithelial cells has been more difficult, requiring innovative strategies to develop such model systems (191–193). During persistent EBV infection of the HK-1 and NP69 human nasopharyngeal epithelial cell lines, signaling pathways including STAT3 and NF-κB are activated compared with uninfected cells, resulting in transcriptional upregulation of downstream targets, including SOCS1 and SOCS3 (169). During EBV infection of human PBMCs, the viral Zta or ZEBRA protein stimulates SOCS3, thereby downregulating JAK/STATs involved with IFN-α/β positive feedback signaling (12).

KSHV (HHV-8), an oncogenic γ-herpesvirus, is the etiological agent of Kaposi's sarcoma (194), a neoplasm of endothelial cells that is characterized by dysregulated angiogenesis and massive inflammation, found primarily in patients with HIV/AIDS (195). During latency, KSHV expresses latency-associated nuclear antigen (LANA) that contains a virally-encoded SOCS box motif, which binds to host cell ubiquitination machinery and flags target proteins including tumor suppressor p53 for proteasome degradation (196). Not only does KSHV encode its own SOCS box-containing protein, it also indirectly induces host SOCS3 in endothelial cells. When infected with KSHV, immortalized human TIME dermal microvascular endothelial cells (DMVECs) significantly induce SOCS3 over uninfected cells or cells infected with UV-inactivated virus at 24, 48, and 96 hpi (197). Like other herpesviruses, KSHV also encodes many proteins homologous with host proteins as well as its own viral-encoded microRNA sequences (195). KSHV-encoded microRNA miR-K12-3 and miRK-12-7 stimulate IL-6 and IL-10 in RAW264.7 mouse macrophages and human myelomonocytic leukemia MM6 cells (198). KSHV-infected primary human endothelial cells repress neutrophil recruitment through stimulation of host IL-6 and SOCS3 (13), and SOCS3 stimulation also suppresses MHC II expression on these cells by suppression of IFN-γ/STAT1 signaling and the downstream class II transactivator (CIITA) (170). Therefore, KSHV and other herpesviruses contain multiple strategies to evade immune surveillance, including stimulation of host SOCS1 and/or SOCS3 by multiple mechanisms.

### Animal Herpesviruses and SOCS1 or SOCS3

MDV (GaHV-2) in the *Mardivirus* genus is an oncogenic α-herpesvirus of chickens. MDV is the etiological agent of Marek's disease, characterized by immunosuppression, neurological disorders, and CD4^+^ T-cell lymphoma with subsequent solid tumors (115, 116). Transmission occurs through inhalation or ingestion of contaminated dust and dander from feather follicle epithelium (199) of the skin of infected chickens. The virus infects many cell types, including lymphocytes, which disseminate through the blood to various organs, including the thymus and spleen (115, 116). Analyses of whole genome arrays have shown that 2–4 days following systemic MDV infection of chickens, SOCS1 and SOCS3 are stimulated in thymus and spleen tissues, with greater upregulation occurring in chicken strains that are more susceptible to MDV (15). Transcriptional stimulation of host SOCS1 and SOCS3 was also found in skin samples of MDV-infected chickens at 20 and 30 days post-infection (19). The specific effects of SOCS1 and/or SOCS3 stimulation during MDV infection are yet unknown.

PRV (SuHV-1) is a *Varicellovirus* endogenous to swine but can infect many different animal and cell types. It therefore has been widely used in various animal model systems, including as a neural tracer (200). In a recent study using PRV infection of RAW264.7 mouse macrophages as an oxidative stress model to measure the antioxidant qualities of *Dunaliella salina* alga extract, it was incidentally reported that PRV induces expression of SOCS3 in these cells at 12 and 24 hpi (20). To our knowledge, thus far the impact of SOCS3 stimulation on PRV infection or pathology remains unknown, as does the effect of PRV on SOCS1 expression.

Mouse-specific salivary gland virus (201, 202), now called MCMV, is in the *Muromegalovirus* genus of the β-herpesvirus subfamily. It frequently is used in experimental mouse models and has contributed greatly to our understanding of infection and pathogenesis of its human-specific counterpart, HCMV (108, 203). HCMV and MCMV both establish latency in circulating monocytes and bone marrow cells (129). SOCS1 and SOCS3 are stimulated very early after *in vitro* MCMV infection of bone marrow macrophages (BMM) (11) as well as IC-21 mouse macrophages and mouse embryonic fibroblast (MEF) cells (18). This stimulation and its temporal patterns are dependent on host cell type and on the mouse strain (C57BL/6 or BALB/c) used for propagation of the MCMV stocks (18). In addition to these *in vitro* models, we have observed in our laboratory that after intraocular (subretinal) MCMV inoculation of immunocompromised mice during experimental MCMV retinitis, SOCS1 and SOCS3 mRNA (16, 23) and protein (21) are upregulated in retinitis-susceptible eyes. As a major topic of focus in this review, the effects of SOCS1 and/or SOCS3 stimulation in this model are discussed in greater detail in a subsequent section of this review.

MHV-68 (or γHV-68) of the *Rhadinovirus* genus natively infects rodents such as mice and voles (204, 205). Because of its genomic and physiologic similarities with both EBV and KSHV, MHV-68 infection of mice is a useful animal model to study pathogen-host interactions of these human γ-herpesviruses (206, 207). It persistently infects lung epithelial cells and establishes latency in B cells, macrophages, and DCs (208). In yet another demonstration of cell type specificity, SOCS1 mRNA and protein are induced upon MHV-68 infection of mouse BMMs and RAW264.7 mouse macrophages, but not MLE-12 mouse lung epithelial cells, NIH3T3 fibroblasts, or MEF cells (22). Transcription of viral genes is likely required for SOCS1 stimulation as UV-inactivation of the virus abrogates this effect. Viral induction of the TLR3/NF-κB pathway induces SOCS1, which then inhibits the antiviral effects of IFN-γ through inhibition of pSTAT1, resulting in increased viral titers (22). Suppression of SOCS1 during MHV-68 infection restores the antiviral qualities of IFN-γ signaling (22). None of these cell types produced SOCS3 stimulation during MHV-68 infection.

### Alphaherpesviruses and SOCS2

In addition to these findings with SOCS1 and SOCS3, a few studies also explore the effects of SOCS2 during α-herpesvirus infection. Following intracranial injection with HSV-1, SOCS2-deficient mice are more resistant to HSV-1 encephalitis, neuroinflammation, and immune cell infiltration to the brain compared with wild type C57BL/6 mice (171), suggesting that SOCS2 contributes to the severity of this disease. HSV-2, the causative agent of genital herpes, has long been debated to have a putative involvement in oncogenesis, particularly as a cofactor in cervical cancer, but this remains unproven (209). In LTEP-α-2 and SPC-α-1 human lung cancer cell lines experimentally infected with HSV-2, the virally-encoded microRNA Hsv2-miR-H9-5p targets and inhibits SOCS2, thereby driving experimental tumor metastasis in these cell lines (172). BHV-5 in the *Varicellovirus* genus natively infects cattle but can establish productive infection in rabbits and mice, which are frequently used as animal models to study neurological disease caused by this virus (173). Unlike HSV-1 infection, infection with BHV-5 exacerbates meningoencephalitis in SOCS2-knockout mice compared with wild type animals (173), suggesting a protective role during intracranial BHV-5 infection. Although it remains unknown whether HSV-1 or BHV-5 stimulates or dampens host SOCS2 expression in these models, SOCS2 nevertheless plays a multivariate role in the pathologies of these herpesviruses.

## Cytomegalovirus Retinitis and SOCS

Despite the development of antiretroviral therapies to treat HIV infection, AIDS-related HCMV retinitis remains a major sight-threatening disease worldwide (110, 152, 180–183). Understanding the pathogenesis of this disease is essential for developing new, safe, and effective treatments for its prevention or management in the clinical setting, yet much remains unknown about the virologic and immunologic mechanisms contributing to its pathology. The pathogenesis of AIDS-related HCMV retinitis involves the complex orchestration of cytomegalovirus infection during AIDS-mediated progressive destruction of the immune system, within the context of retinal cells in the eye.

Vision is facilitated by a complex system whose gross anatomy, microanatomy, biophysical, and biochemical properties are critical to its function. Disruption of any one of thousands of components of this system could lead to visual impairment or blindness. Light first encounters the cornea, which acts as a powerful lens to focus light through the liquid-filled anterior chamber, through the aperture of the pupil, and into the crystalline lens. The lens focuses light with greater precision through the viscous vitreous gel and onto the parfait-like layers of the neurosensory retina at the back of the eye. Photoreceptors in the retina detect photons of light and transmit signals through first-order, second-order, and third-order neurons into ganglion cell axons that exit the eye as the optic nerve. The specialized neuronal cells of the retina are supported by networks of Müller cells, astrocytes, and microglia, as well as by the RPE, a specialized layer of phagocytic, multifunctional epithelial cells (210). As part of the posterior segment of the eye and an extension of the brain, the retina is considered an immune-privileged site (211) primarily because it does not elicit a typical inflammatory immune response to the introduction of antigens (212, 213). Thus, irreplaceable neuronal tissue is somewhat protected from the damaging effects of inflammation and immunopathogenesis.

### AIDS-Related HCMV Retinitis

When the immune privilege of the ocular compartment fails, inflammation causing severe immunopathogeneses and permanent, sight-threatening damage may occur, as in the case of AIDS-related HCMV retinitis. Prior to the era of antiretroviral therapies, this progressive necrosis of the retina is estimated to have occurred in ~30% of HIV/AIDS patients with CD4^+^ T-cell counts fewer than 50 cells/μL blood (25, 180, 181, 214–216). Antiretroviral therapies targeting HIV have greatly reduced the number of new cases of AIDS-related HCMV retinitis in developed countries (151, 180) but have failed to eliminate them completely (215). This disease therefore remains a significant clinical problem worldwide.

Although HCMV is ubiquitous in the population and relatively mild as an infectious disease of immunocompetent individuals, it can become a severe opportunistic pathogen during the immune suppression that occurs when HIV infection progresses to AIDS. It is likely that during AIDS-related HCMV retinitis, HCMV reactivates from latency and travels to the eye hematogenously within monocytes or macrophages, as ophthalmoscopic examination of the retina reveals the characteristic foci of dense retinal whitening that follow retinal blood vessels and may be accompanied by hemorrhage (151). Failure to treat AIDS-related HCMV retinitis results in blindness of most or all of the affected eye, usually followed within 1 year by vision loss in the contralateral eye (110, 152, 180–183). The mechanisms of blindness involve destruction of the retina itself, retinal detachment, or a uveitis that can occur with reconstitution of the immune system associated with well-tolerated antiretroviral therapies (immune recovery uveitis, IRU) (151, 180). Current treatment strategies for HIV/AIDS patients presenting with HCMV retinitis target HCMV replication through lifelong administration of antiviral drugs such as ganciclovir, cidofovir, or foscarnet that can control but not eradicate the virus, slowing but not reversing HCMV-induced ocular damage (217–221). Unfortunately, frequent administration of these drugs has led to an increase in drug-resistant strains of HCMV (222). Vaccination has been one of the most effective methods for controlling other problematic infectious diseases, but attempts to engineer a suitably efficacious vaccine against HCMV thus far have been unsuccessful (223, 224).

### Mouse Models of Experimental Cytomegalovirus Retinitis

Because the species-specificity of HCMV precludes its ability to establish productive infection in animal models or cells (225), MCMV is commonly substituted in research laboratories to investigate cytomegalovirus infection and pathogenesis in mouse models (108, 203) because of high face validity and predictive validity (27). Such research with MCMV has significantly improved our collective understanding of HCMV characteristics and pathogeneses, including the involvement of immune cell types such as CD8^+^ T cells and NK cells in controlling infection (110).

As with humans and HCMV, immunologically normal mice are generally resistant to MCMV retinitis (24, 147, 226, 227), depending on mouse strain (228, 229), viral load, and route of viral inoculum (230–232). Establishment of an immune-suppressed state together with delivery of a substantial amount (10^4^ plaque forming units, pfu) of MCMV directly into the subretinal space of the eye overcomes this resistance, consistently manifesting high frequencies (75–100%) of experimental MCMV retinitis (29, 150, 230) in a manner dependent upon viral load (230) and mouse strain (24, 150, 228–233). Two successful immunosuppression strategies to achieve susceptibility to MCMV retinitis include systemic delivery of corticosteroid drugs (150, 230, 234) or a mixture of mouse-specific retroviruses designated lymphoproliferative-bone marrow 5 (LP-BM5) (235, 236) that induces MAIDS after 8–10 weeks in C57BL/6 mice (26, 237, 238).

The strain of mouse used during experimental MCMV retinitis studies impacts susceptibility to MCMV infection and to the MAIDS-producing LP-BM5 retrovirus mixture. BALB/c mice are more susceptible than C57BL/6 mice to systemic MCMV infection (228, 231, 239–242), and this appears to affect the incidence of experimental retinitis in the corticosteroid model. During corticosteroid-induced immune suppression, the frequency of MCMV retinitis in BALB/c mice is about 90% (150), compared with 50% in C57BL/6 mice (23, 233). BALB/c mice, however, are more resistant than C57BL/6 mice to the induction of MAIDS by LP-BM5 (26, 243), as C57BL/6 mice reach late-phase MAIDS within 10 weeks whereas a year or longer is required for BALB/c mice to progress to late-stage MAIDS. For this reason, although BALB/c mice are generally used for experimental MCMV retinitis models with corticosteroid-induced immune suppression, C57BL/6 mice are used for MAIDS models. Importantly, the frequency of experimental MCMV retinitis after subretinal MCMV injection in C57BL/6 mice with MAIDS is 80–100% (24, 226, 227), comparable with the frequency in drug-immunosuppressed BALB/c mice (150).

Just as later stages of AIDS in humans correlates with greater susceptibility to HCMV retinitis, so mice with late-stage MAIDS at 10 weeks (MAIDS-10) are more susceptible to MCMV retinitis than mice with early- or mid-stage MAIDS around 4 weeks (MAIDS-4). Importantly, SOCS1 and SOCS3 are highly stimulated following subretinal MCMV infection in the retinitis-susceptible eyes of MAIDS-10 mice, but not in the MCMV-infected retinitis-resistant eyes of MAIDS-4 mice (16, 21). In C57BL/6 mice with corticosteroid-induced immune suppression, however, subretinal MCMV infection does not significantly alter SOCS1 or SOCS3 protein expression and only mildly stimulates SOCS3 mRNA (23). To our knowledge, the effect of subretinal MCMV infection on SOCS1 and SOCS3 expression in the eyes of BALB/c mice during corticosteroid-induced immune suppression has not been reported to date.

In the absence of MCMV infection, these two different techniques to accomplish immune suppression also differ in their types of dysfunctional immune cells and the timing of immune cell demise (23). One of the major differences between these models is the number and function of macrophages. MAIDS, without MCMV infection, causes reduced Mac1^+^ (CD11b^+^) macrophage population percentages and activation frequencies at MAIDS-4 (237, 244), with increased macrophage numbers between MAIDS-8 and MAIDS-12 (245). Macrophage populations in MAIDS mice are driven toward an alternatively-activated proangiogenic phenotype that is between classically-activated M1 and alternatively-activated M2. They have decreased TNF-α and IFN-α production but increased IL-1β and IL-6 production in response to LPS (246, 247). By contrast, corticosteroids such as methylprednisolone acetate, in the absence of MCMV infection, very quickly suppress or destroy most of both the innate and adaptive immune systems, including macrophages (248). Whatever macrophages remain tend to be driven toward the M2 alternatively-activated phenotype, in a similar manner as macrophages exposed to IL-4, and they avidly produce IL-10, but not TNF-α, IL-1, or IL-6 (134, 135). Therefore, whereas MAIDS mice experience a functional change in macrophage phenotype after weeks (245–247), drug-induced immune suppression decreases macrophage populations within days (248). Corticosteroids also decrease the overall number and function of CD4^+^ and CD8^+^ T cells [~93% depletion, (234, 248, 249) and generally dampen the immune response by suppressing the expression, release, and/or function of inflammatory cytokines such as IFN-γ TNF-α, and IL-2 (249). This rapid, acute decline of the immune system is not observed during MAIDS, which slowly progresses through distinct phases of immune cell dysfunction. Whereas corticosteroid treatment causes apoptosis in leukocytes and lymphocytes therefore decreasing the overall number of these populations (248, 249), MAIDS causes aberrant proliferation of B and T lymphocytes (250, 251) that results in increases in these cell populations coupled with retrovirus-induced cellular dysfunction (26, 251, 252). By late-stage MAIDS, NK cells (253), and neutrophils (254) are also dysfunctional, and macrophage phenotypes are irregular (245–247).

Throughout the many years that these mouse models have been studied, both drug-induced and retrovirus-induced immune suppression strategies during subretinal MCMV infection have contributed to our collective theoretical knowledge of MCMV retinitis and our clinical knowledge of HCMV retinitis. While the drug-induced immune suppression model yields relatively faster results, it bypasses the many nuances and complexities of retroviral immune suppression that the MAIDS model alone bridges to clinical relevance.

### MAIDS-Related MCMV Retinitis and SOCS

AIDS of humans and MAIDS of mice are both caused by species-specific retroviruses and share many immunologic and pathologic features (26, 237). Both syndromes are characterized by progressive generalized lymphadenopathy, polyclonal B-cell activation (250), diminished CD4^+^ T-cell and CD8^+^ T-cell functions (251), and a cytokine shift from a T_H_1 origin to T_H_2-associated cytokines (236, 255, 256). Although profound splenomegaly also occurs in MAIDS mice, this overall increase in splenic cell counts is associated with dysfunctional immune cells (257). By MAIDS-10, B cells (247, 258), CD4^+^ and CD8^+^ T cells (245, 251, 259), NK cells (253), and neutrophils (254) are dysfunctional, and macrophage phenotypes are irregular (245–247). Mice with late-stage MAIDS (8–12 weeks) develop a retinitis at 8–10 days following subretinal MCMV injection that exhibits histopathologic features similar to those found in AIDS-related HCMV retinitis (24, 260), including full-thickness retinitis, cytomegalic cells, and transition zones of histologically normal to necrotic retina. Table 3 summarizes the similarities and differences between the retroviruses causing AIDS or MAIDS, and between HCMV retinitis and MCMV retinitis during each, respectively.

**Table 3:** AIDS-related HCMV retinitis vs. MAIDS-related MCMV retinitis.

	**AIDS-related HCMV retinitis**	**MAIDS-related MCMV retinitis**
**Retrovirus-Induced Immune Suppression**		
Macrophages among targeted cell types	Yes	Yes
Polyclonal B-cell activation	Yes	Yes
Hypergammaglobunemia	Yes	Yes
Splenomegaly	No	Yes
T_H_1-to-T_H_2 cytokine shift	Yes	Yes
Diminished CD4^+^ and CD8^+^ T-Cell:		
Numbers	Yes	No
Functions	Yes	Yes
**Cytomegalovirus Retinitis Histologic Characteristics**		
Foci of cytomegalic cells	Yes	Yes
Hemorrhage	Yes	Yes
Transition zones between normal and necrotic retina	Yes	Yes
Full-thickness retinal necrosis	Yes	Yes

*Reviewed in Jolicoeur (26) and Watson (237)*.

Immunologically normal C57BL/6 mice and MAIDS-4 C57BL/6 mice are resistant to MCMV retinitis (0% frequency). Mice with MAIDS-8 to MAIDS-12, however, are susceptible (80–100%) to MCMV retinitis following subretinal (24, 226, 227), but not systemic (232), MCMV inoculation. Importantly, retinitis susceptibility does not correlate with ocular viral titers, because MCMV replication in the ocular compartment at 6–10 days after subretinal inoculation reaches equivalently high levels (~3 × 10^4^ pfu/eye) in retinitis-resistant MAIDS-4 mice as those in retinitis-susceptible MAIDS-10 mice (227, 261). By comparison, immunologically normal mice receiving the same amount of subretinally-injected MCMV typically produce only ~10^2^ pfu/eye (24). Thus, high intraocular MCMV titers alone are insufficient for retinitis, and susceptibility to intraocular MCMV replication precedes susceptibility to retinitis in this model (227).

Thus far mechanisms of humoral immunity (262), cellular immunity (263, 264), cell death pathways (261), and several cytokines have been studied during onset and development of retinal disease in the MAIDS model of MCMV retinitis. Among the putative SOCS-inducing cytokines examined in this model are TNF-α (227, 261), IFN-α/β and IL-6 (21), IFN-γ (21, 227), IL-2 (265, 266), IL-12 (266), IL-4 (226, 267), IL-10 (267), and IL-17 (16). In addition, SOCS1 and SOCS3 are highly stimulated following MCMV infection in retinitis-susceptible MAIDS-10 eyes, but not MCMV infected retinitis-resistant MAIDS-4 eyes (16, 21). In MAIDS-10 eyes with MCMV retinitis, SOCS1 and SOCS3 are produced by infiltrating macrophages and granulocytes, as well as resident microglia and Müller cells (21). Uninfected bystander cells as well as MCMV-infected cells of the retina also abundantly produce SOCS1 and SOCS3 (21), a phenomenon that also has been reported in MCMV-infected IC-21 macrophages (18) and in HCMV-infected monocyte-derived DCs (14). Systemic MCMV in immunocompetent mice without MAIDS moderately stimulates splenic SOCS1 transcripts and SOCS-inducing cytokines IFN-γ and IL-6, but this stimulation decreases in amplitude as MAIDS progresses (21). Furthermore, there is a decreased intraocular stimulation of SOCS1 and SOCS3 during experimental MCMV retinitis during corticosteroid-induced immune suppression that correlates with reduced severity of retinitis (23). Thus, during *in vivo* MCMV infection, substantial and extended SOCS1 and SOCS3 stimulation appears only in the eye (21) and is correlated with more severe MCMV retinitis (23). Stimulation of pro-inflammatory and antiviral cytokines such as TNF-α and IFN-γ in the eyes of mice with severe MAIDS-related MCMV retinitis fails to control viral replication, but concurrent stimulation of anti-inflammatory cytokines like IL-10 and IL-4 is not sufficient for protection against ocular immunopathogenesis in this disease model (21). Although many questions remain, SOCS1 and/or SOCS3 may play promising roles in the balance of this phenomenon, potentially revealing themselves as novel therapeutic targets to improve the management and/or prevention of AIDS-related HCMV retinitis.

### SOCS1 or SOCS3 as Potential Therapeutic Targets During Cytomegalovirus Retinitis

Several strategies for inhibiting or enhancing SOCS1 or SOCS3 gene expression or protein activity in the context of infectious or inflammatory diseases, including over-expression or inhibition gene therapies via viral vectors, have been developed and tested *in vitro* and *in vivo* with promising results, as summarized elsewhere (6). One attractive approach to control the functions SOCS1 and/or SOCS3 includes therapeutic use of small-molecule protein antagonists or mimetics of SOCS1 and/or SOCS3 proteins.

Although stimulation of SOCS1 and SOCS3 during experimental MAIDS-related MCMV retinitis suggests that one or both of these contribute to the severity of the disease, at this time it remains unknown whether SOCS1 and/or SOCS3 inhibition or overexpression would improve the clinical outcome of AIDS-related HCMV retinitis. If SOCS1 and/or SOCS3 contribute to the pathogenesis of this disease, then their inhibition in HIV/AIDS patients with HCMV retinitis could prevent further damage to affected eyes and/or protect the contralateral eye from vision loss. One such SOCS-sequestering small synthetic peptide is pJAK2[1001–1013] (LPQDKEYYKVKEP), which includes the phosphorylated activation loop of JAK2 (44, 268) and antagonizes both SOCS1 and SOCS3. This peptide has shown efficacy against HSV-1 infection in keratinocytes (166) and protects against lethal doses of vaccinia virus, encephalomyocarditis virus, and influenza A virus in mice (269, 270). Because SOCS1 and SOCS3 dampen the ability of cytokines to propagate effective signals within their target cells, inhibition of SOCS1 and/or SOCS3 coupled with immunotherapy treatments such as antiviral IFNs (271) could improve the efficacy of such treatments.

It remains a possibility that the immunosuppressive effect of SOCS1 and/or SOCS3 may play a protective role against a potential immunopathology of experimental MCMV retinitis or AIDS-related HCMV retinitis. If overexpression of SOCS1 and/or SOCS3 reduces retinitis severity, SOCS1 and/or SOCS3 mimetic peptides or overexpression treatment strategies could be efficacious against this disease, as with experimental autoimmune uveitis (EAU) (272, 273). This seems to be the case for HSV-1 infection in the eye, where the role of SOCS1 during HSV-1 infection appears to be protective despite *in vitro* HSV-1 infection stimulating SOCS1 and SOCS3 very early to increase viral load and cytopathology in different cell types (166, 175). In transgenic rats overexpressing SOCS1 in the retina, however, intraocular HSV-1 (McKrae strain) infection is reduced or delayed compared with wild type rats (274). These SOCS1-overexpressing rats bred to a Lewis strain background also display reduced severity during interphotoreceptor retinoid binding protein (IRBP) antigen-induced (retina-specific) EAU (275). In a mouse model of IRBP antigen-induced EAU, treatment with the cell-penetrating SOCS1-KIR-derived peptide (272, 273) reduces severity of disease. EAU is also less severe in mice containing a conditional SOCS3 knockout in CD4^+^ T-cells (276). The anti-inflammatory role of SOCS1 and/or SOCS3 functioning with cell-type-specificity within the complexity of the eye may therefore protect the precious cells of the retina during immunopathologies such as intraocular HSV-1 infection or autoimmune uveitis. Further studies utilizing knockdown or overexpression of SOCS1 or SOCS3 would elucidate this possibility for experimental MCMV retinitis and/or AIDS-related HCMV retinitis.

## Concluding Remarks

Host manipulation strategies among herpesviruses, diverse and redundant, share many similarities, such as stimulation of host SOCS1 and/or SOCS3. The virologic, immunologic, and pathologic effects of SOCS1 or SOCS3 stimulation during herpesvirus infection frequently depend on cell type, virus strain, and host or host organ system. Such parameters reflect the complexities of the diverse cells and organ systems directly or indirectly involved with herpesvirus infection, disease, and latency. Although it remains unclear whether viral stimulation of SOCS1 and/or SOCS3 is protective or pathogenic in the eye during AIDS-related cytomegalovirus retinitis, these host proteins may yet prove useful therapeutic targets for treatment or prevention of this sight-threatening disease, as well as other disease of herpesvirus etiology.

## Author Contributions

CA composed and RD conceptualized this review. Both authors contributed to manuscript revision and approved the submitted version.

### Conflict of Interest Statement

The authors declare that the research was conducted in the absence of any commercial or financial relationships that could be construed as a potential conflict of interest.
